# Environmental Risk and Meningitis Epidemics in Africa

**DOI:** 10.3201/eid0910.030182

**Published:** 2003-10

**Authors:** Anna M. Molesworth, Luis E. Cuevas, Stephen J. Connor, Andrew P. Morse, Madeleine C. Thomson

**Affiliations:** * Liverpool School of Tropical Medicine, Liverpool, United Kingdom; † University of Liverpool, Liverpool, United Kingdom

## Abstract

Epidemics of meningococcal meningitis occur in areas with particular environmental characteristics. We present evidence that the relationship between the environment and the location of these epidemics is quantifiable and propose a model based on environmental variables to identify regions at risk for meningitis epidemics. These findings, which have substantial implications for directing surveillance activities and health policy, provide a basis for monitoring the impact of climate variability and environmental change on epidemic occurrence in Africa.

Epidemics of meningitis occur worldwide. However, the “meningitis belt” of Africa’s Sahel region has the greatest incidence of the disease, with large epidemics of predominantly group A meningococci; the endemic levels found in this region would be considered epidemic elsewhere. Although factors predisposing populations to meningitis epidemics are poorly understood, population susceptibility, introduction of new strains, poor living conditions, and concurrent infections have all been implicated. Epidemics occur throughout Africa in the dry season, coincide with periods of very low humidity and dusty conditions, and disappear with the onset of the rains, suggesting that these environmental factors may also play an important role in the occurrence of the disease (*1*–*3*).

Lapeyssonnie (*4*) observed in 1963 that epidemics largely occurred in a semi-arid zone south from the Sahara, with 300–1,100 mm mean annual rainfall, and Cheesbrough et al. (*5*) suggested in 1995 that areas that are humid throughout the year have low disease rates. In West Africa, Waddy (1958) described an area that suffered epidemics as having “… only one definable frontier, the junction of the forest…with the savanna…, when there is an abrupt change from a permanently humid climate to one with a severe dry season” (*6*). Epidemics have been rarely reported from the humid forested or coastal regions, even when neighboring areas are severely affected.

With current methods, the timing of future outbreaks is unpredictable, and tools that identify the key environmental factors associated with areas prone to meningitis epidemics would help us to understand the basis for these outbreaks and eventually optimize prevention and control activities. We describe a model that predicts the probability, based on environmental information, of an area experiencing an epidemic of meningitis.

## Methods

### Epidemiologic Data

Details of all known meningitis epidemics occurring before 2000 in countries comprising continental Africa were compiled from information documented in the published literature and unpublished institutional reports at the end of June 2001. All epidemics reported in the medical literature were identified in PubMed’s online database of medical literature (United States National Library of Medicine, available from: URL: http://www.ncbi.nlm.nih.gov/PubMed/) through manual searches and by cross-referencing publications. We obtained unpublished information from Web searches or directly from international and national organizations involved in disease control and humanitarian aid (*1*).

Epidemics reported at the provincial (second administrative level) and district level were located by using administrative boundaries available from the U.S. Geological Survey EROS Data Centre Africa Data Dissemination Service, Sioux Falls, SD (available from: URL: http://edcintl.cr.usgs.gov/adds/). Locations of villages or towns were verified by using the National Imagery and Mapping Agency’s GEONet gazetteer (available from: URL: http://www.nima.mil/), and historical reference atlases and maps contained in the original reports were mapped to the current administrative boundaries by using ArcView 3.1 geographic information system (GIS) (ESRI, Redlands, CA). We assumed that events reported at the provincial level affected all constituent districts and excluded epidemics reported only at the national level. Events affecting parts of any one country in one epidemic season were considered as one epidemic. We then classified the 3,281 districts of Africa as ever or never having experienced a documented epidemic of meningitis. In this analysis, no attempt was made to distinguish between epidemics of different scales.

### Environmental Data

Environmental information for the African continent was obtained from a variety of sources. In this analysis, we restricted data to variables available in the public domain with digital grid–based uniform continental coverage, which we considered important (*7*). Variables included monthly means (long-term averages) of absolute humidity, absorbing aerosols (dust) and rainfall, and land-surface maps of land-cover type and population density (Table 1). All data grids were incorporated into the GIS.

**Table 1:** Characteristics of the environmental variables

Variable	Temporal resolution	Time period	Resolution of grid squares	
Interpolated meteorologic station dataa				
Average daily mean absolute humidity	Mean monthly	1961–1990	0.5° lat x 0.5° long (nominal 50 km)	
Average daily rainfall	Mean monthly	1961–1990	0.5° lat x 0.5° long (nominal 50 km)	
Remotely sensed satellite datab				
Average daily aerosol index (dust)	Mean monthly	1980–1999	1.0° lat x 1.25° long (nominal 100 km)	
Digital maps				
Land-cover typec		1992–1993	1 x 1 km	
Population densityd		1990	0.042° lat x 0.042° long (nominal 4 km)	

^a^Mean monthly climate averages (1961–1990) for absolute humidity and rainfall (the former derived from vapor pressure and mean temperature) were obtained from the Climate Research Unit, University of East Anglia, UK (available from: URL: http://ipcc-ddc.cru.uea.ac.uk/). ^b^Dust was obtained as monthly aerosol index coverages from the U.S. National Aeronautics and Space Administration (NASA), Goddard Space Flight Center, Maryland (available from: URL: http://www.gsfc.nasa.gov/), excluding the period May 1993 to June 1996 for which data are not available. ^c^Land-cover type was obtained from the USGS NASA PATHFINDER 1km project (available from: URL: http://edcwww.cr.usgs/gov/landdaac/1KM/).  ^d^U.S. Geologic Survey 1990 population density forecasts (available from: URL: http://grid2.cr.usgs.gov/globalpop/africa/).

**Figure 1:**
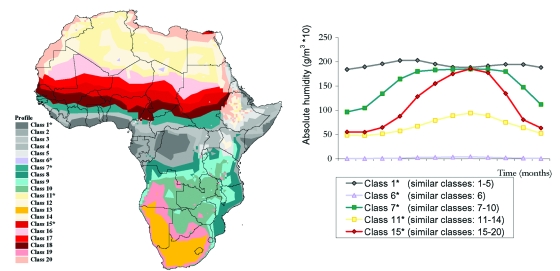
Ecologic variation in the seasonal profile of absolute humidity. a) spatial variation in profile class; b) representative profile class.

To collect the seasonal variation in climate while reducing the number of explanatory variables without loss of information, we reprocessed the monthly means of each variable to create a single surface comprising categories representing unique seasonal profiles. This reprocessing involved submitting the monthly means to a principal components analysis followed by a clustering procedure in which we grouped regions with a similar seasonal profile using ADDAPIX software (version 2.05, S. Griguolo, University of Venice, Italy, available from: URL: http://metart.fao.org/T_I/GBR/Tools/Eaddapix.htm). This software performs spatial and temporal analyses of time-series data in continuous grid-based surfaces (*8*). The use of seasonal profiles for describing the climate of an area is widely used in crop monitoring in agriculture and has been used in modeling malaria prevalence in Gambia (*9*). The profile surfaces were then imported into ArcView 3.1 (ESRI). The seasonal absolute humidity profile is shown in Figure 1.

For every district, a value was extracted in GIS, representing those grid cells of each profile surface contained within the district boundary. These values comprised the most common seasonal profile class for each variable (absolute humidity, dust, and rainfall), the most common land-cover type, and the geometric mean population density.

### Analysis

A logistic regression analysis was used to identify associations between a district ever or never having experienced an epidemic and the environment by using SPSS 11.0 software (SPSS Inc., Chicago, IL). Explanatory variables were first investigated individually and then entered stepwise into a forward conditional multiple regression analysis. Because of the need to reduce colinearity, we did not analyze environmental variables with similar geographic distributions together in the same model. The final model was based on the simplest approach and a combination of variables that best predicted the distribution of epidemics. This model was created with and without weighting for the inverse size of the district to assign less weight to larger districts, which may have been more prone to ecologic variation and therefore inadequately represented by a single value. The probability of each district ever having had an epidemic was predicted by using the model. These probabilities were grouped into risk categories and mapped, and the estimated total population was extracted in GIS derived from population density forecasts (U.S. Geological Survey, 1990).

The sensitivity and specificity of the model were assessed by examining the agreement between predicted and observed epidemic experience by using a receiver-operator characteristics (ROC) curve to select the optimal probability cutoff values on which predictions are based (*10*). The dataset was then split at random into two parts containing approximately 60% and 40% of the 3,281 districts. The model was recreated with the 60% dataset, using the same variables as above and used to predict the risk for epidemics in the remaining 40% (the validation set). We repeated this process 10 times and compared the mean sensitivity and specificity of the validation set with the model derived from the entire dataset. Residuals resulting from the differences between the observed and predicted risks were also calculated and mapped to establish whether errors in the model were randomly distributed, thereby supporting the validity of the model throughout the continent.

## Results

**Figure 2:**
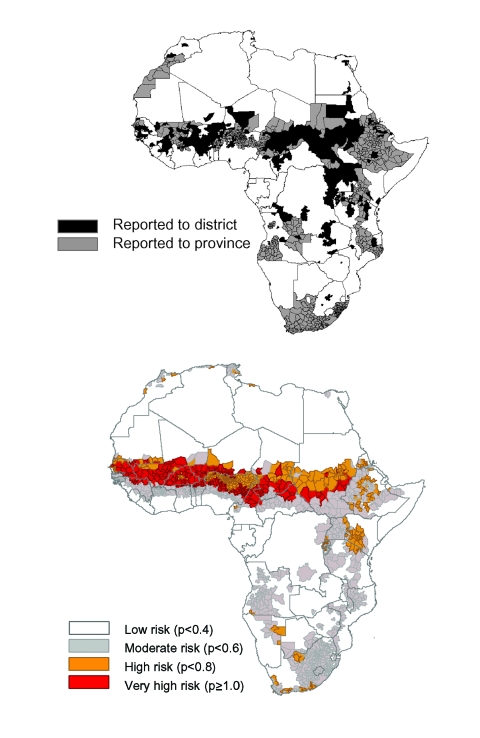
Distribution of districts where epidemic meningococcal meningitis are likely to occur. a) observed distribution of meningitis epidemics (1841–1999). b) fitted model of observed meningitis epidemics based on environmental variables.

The earliest documented meningitis outbreak in Africa affected a French garrison in 1841 in Algiers (*11*), and >425 epidemics were documented at the subnational level for the next 158 years. These epidemics affected at least 1,231 (38%) of the 3,281 continental districts. Supporting our earlier findings, epidemics were not evenly distributed across the continent, instead affecting mainly districts in the Sahel and south of this region and extending from northern Uganda and the eastern part of Democratic Republic of Congo, through the Great Lakes and the Rift Valley regions to Malawi and northeastern Mozambique, and from northeastern Mozambique west and south to include other parts of southern Africa (Figure 2a) (*1*).

Absolute humidity, dust and rainfall profiles, land-cover type, and population densities were independently associated with the location of epidemics. However, we found that absolute humidity profile and land-cover type were the best predictors in the final multivariate model. Certain dust profiles and population density made only marginal difference to the performance of the model and were excluded to maintain simplicity. In addition, absolute humidity and rainfall profiles predicted similar risks in geographic locations in north and west Africa, but a model including the former performed better for the entire continent than one including rainfall; for this reason, we kept absolute humidity as a variable. Weighting for district size did not improve the performance of the model and was discarded.

The model, which is based on the absolute humidity profile and land-cover type, is described in terms of its baseline characteristics, estimated coefficients, standard errors, and contribution of the variables in Table 2. The risk map for epidemic experience in Africa derived from this model is presented in Figure 2b. The most important factor associated with the distribution of epidemics was humidity. Areas without a marked distinction between wet and dry seasons were less likely to have had epidemics than those with contrasting seasons. The areas without distinction between wet and dry seasons include deserts and the humid parts of coastal and central Africa, much of which are forested, and the areas with contrasting seasons comprise the semiarid savannah and grasslands of the Sahel and east and southern Africa. Surface maps of Africa demonstrated a close correspondence between humidity and land-cover types in these regions. The model also showed that, having accounted for the effects of humidity, sparsely vegetated and barren regions, areas of woodland mosaic, and shrub land were less likely than other regions to have ever had an epidemic. The Sahel, which has a prolonged dry season with low humidity was identified as the area with the greatest risk (p>0.6). Peripheral regions along its southern borders, where the dry season is shorter and less extreme, carry a moderate risk (p>0.4). The peripheral region extends from southern Sudan and Ethiopia to the Great Lakes and Rift Valley regions and parts of southern Africa peripheral to desert areas.

**Table 2:** Baseline characteristics, estimated coefficients, and standard errors for the model

Variable	Epidemic experience (n districts)^a^		Multi-variable analysis		
	Ever (%)	Never	β	SE	
Absolute humidity profile^b^					
Class 1	15 (3)	527	Reference		
Class 2	9 (6)	153	0.59	0.44	
Class 3	19 (8)	228	1.00^c^	0.36	
Class 4	43 (47)	48	3.48^c^	0.35	
Class 5	143 (61)	93	3.97^c^	0.31	
Class 6	0 (0)	2	–2.44	15.73	
Class 7	118 (47)	132	3.29^c^	0.30	
Class 8	40 (28)	102	2.40^c^	0.33	
Class 9	106 (48)	117	3.31^c^	0.31	
Class 10	46 (22)	161	2.12^c^	0.32	
Class 11	16 (29)	39	3.34^c^	0.43	
Class 12	1 (11)	8	2.17^d^	1.13	
Class 13	90 (48)	97	3.30^c^	0.32	
Class 14	44 (52)	40	4.00^c^	0.36	
Class 15	178 (74)	64	4.46^c^	0.32	
Class 16	7 (37)	12	3.61^c^	0.59	
Class 17	25 (54)	21	3.99^c^	0.43	
Class 18	181 (80)	46	4.82^c^	0.32	
Class 19	105 (56)	84	3.60^c^	0.31	
Class 20	46 (39)	73	3.45^c^	0.35	
Land-cover type					
Savanna	646 (39)	1006	Reference		
Dry land crop land/pasture	44 (39)	68	–0.38	0.23	
Irrigated crop land/pasture	0 (0)	9	–7.02	7.14	
Cropland/grassland mosaic	198 (53)	174	–0.07	0.14	
Cropland/woodland mosaic	4 (4)	105	–1.97^c^	0.56	
Grassland	123 (67)	61	0.36	0.19	
Shrub land	66 (34)	127	–0.52^c^	0.19	
Urban	3 (43)	4	0.32	0.85	
Broadleaf deciduous forest	44 (39)	68	0.17	0.23	
Evergreen broadleaf forest	37 (13)	248	0.07	0.24	
Water bodies	20 (38)	33	–0.07	0.33	
Forest wetland	0 (0)	24	–2.82	4.55	
Barren/sparsely vegetated	46 (28)	120	–1.07^c^	0.24	
Variable removed^e^	Log likelihood	Change in –2 likelihood	df	Significance of change	
Absolute humidity profile	–2045.937	877.963	19	<0.001	
Land-cover type	–1642.564	71.217	12	<0.001	

^a^Excludes five districts for which environmental data were unavailable (in Sinai, Egypt, and Pemba, Tanzania). ^b^p value <0.01. ^c^p value <0.05. ^d^See Figure 1 for description of profile classes. ^e^Model if term removed.

**Figure 3:**
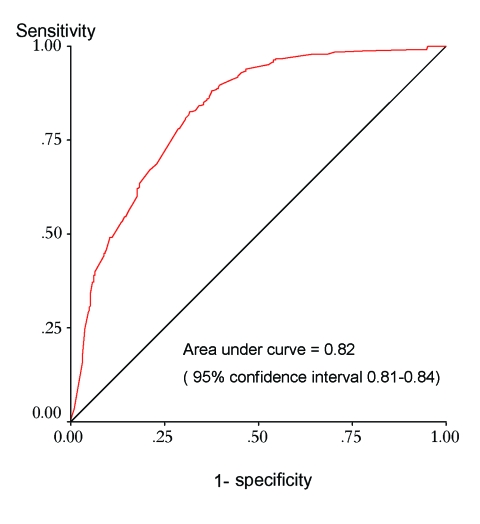
Receiver-operator characteristics curve for the model.

The ROC curve used to describe the performance of the model in terms of its sensitivity and specificity at various cutoffs; its overall accuracy independent of a single probability cutoff is shown in Figure 3. The model can discriminate between districts that have experienced epidemics and those that have never been affected. When the values for randomly selected districts were entered into the model, the epidemic risk assigned was higher for districts with epidemics than for those without in 82% of the cases. The areas identified by the model coincide to a large extent with the areas observed to have experienced epidemics. By using a probability cutoff value of >0.4 for predicting epidemic experience, the model had a sensitivity and specificity of 83% and 67%, respectively; these statistics were confirmed in the validation process (Table 3). The map of residual risk unaccounted for by the model had a random distribution, as expected for a model that worked well across the continent (not shown). According to the model, 7% of the population of Africa live in very high-risk areas, 17% in high, and 27% in moderate (based on 1990 estimates), corresponding to 44, 102, and 162 million people, respectively (Table 4).

**Table 3:** Performance of the model for predicting epidemic experience at the district level^a^

Epidemic experience	Observed		
Predicted	Ever	Never	Total districts
Ever	1,022	682	1,704
Never	209	1,363	1,572
Total districts	1,231	2,045	3,276
Model	Sensitivity % (95% CI)		Specificity % (95% CI)
Final (100%)	83 (81 to 85)		67 (65 to 69)
Mean validation (40%)	84 (80 to 87)		65 (62 to 69)

^a^Excludes five districts for which environmental data were unavailable; CI, confidence interval.

**Table 4:** Population residing in districts at risk for meningitis epidemics in Africa

Risk	No. of districts	1990 population in millions (%)
Low	1,572	291 (49)
Moderate	971	162 (27)
High	482	102 (17)
Very high	251	44 (7)
Not known	5	1 (<1)
Total	3,281	600

## Discussion

This study represents a comprehensive and detailed description of the spatial distribution of meningitis epidemics at the district level in Africa and the first attempt to develop a spatial forecasting model for meningitis epidemics on the basis of the environmental characteristics of the continent considered a priori to be related to the spatial distribution of epidemics. The data have limitations that need to be considered for the proper interpretation of the models. For example, census data for Africa have limited accuracy (*12*), variables derived through remote sensing may only partially capture surface conditions, and data from meteorologic stations in Africa are often incomplete (*13*–*15*). The epidemiologic data span more than a century whereas the environmental and population data are relatively recent. In addition, some epidemics were likely never reported, and small outbreaks and those occurring in the 19th and early 20th centuries are likely to be disproportionately underrepresented. Problems in defining epidemics exist as well, since most reports lack clear or internationally recognized criteria; we had to accept the perception of an increased incidence that prompted outbreak reports. Moreover, the aggregation of local level statistics to an often larger and somewhat arbitrary district level, discrepancies between where people become ill and the location of notifying health facilities, and changes to district boundaries over time may each have resulted in potential loss of specificity. Despite these limitations, major outbreaks were unlikely to have gone completely unreported, and the long-term cumulative distribution of events is likely not misrepresented on a pancontinental scale. While population densities in Africa have increased greatly during the last 150 years and substantial land-use change (particularly in West Africa) is known to have occurred, the model was still able to identify the meningitis belt and areas previously described at risk beyond the Sahel (*2*–*5*,*16*); reports of epidemics occurring since 1999 have coincided with this description (available from: URL: http://www.who.int/disease-outbreak-news/).

The model also has its limitations. We are aware of those imposed on the analysis by spatial relationships in the data, which indicate that the importance of regression effects may be overstated (*17*). No perfect model exists, but one can be developed that is based on the simplest approach and combination of variables that can be used to distinguish between areas with high and low risk of epidemic experience. We were restricted by the limited availability of suitable datasets and chose the simplest combination of variables that best predicted outcome. While this choice is likely to have oversimplified the association between meningitis epidemics and the environment, the model has the advantage of using data that are available in the public domain and, being based on a simple combination of variables, are an important basis on which to develop research and future operational applications in resource-limited settings.

The model indicates not only that absolute humidity profiles and land-cover types can be used to distinguish between areas with high and low risk of epidemics but also that population density and dust may also be implicated. The incidence of meningococcal disease has previously been correlated with dry and dusty conditions in tropical and temperate climates (*18*–*21*). Humidity and land cover were included in the final model for statistical reasons, but dust and population density still have an independent effect and may be important in determining epidemic occurrence (*22*). The potential role of dust in precipitating epidemics is particularly interesting since dustiness in the meningitis belt has increased dramatically since the Sahelian droughts of the 1970s and 1980s. However, how environmental variables interact is unclear and remains the subject of extensive climatologic research. Furthermore, we did not take into account the effect of other nonenvironmental factors likely to be related to epidemics, such as population movement, vaccination coverage, and recent epidemics in the area. A combination of conditions is likely to be necessary for an epidemic to occur, and these nonenvironmental variables are likely to have additional predictive potential and should be considered in further studies.

Risk maps of vector-borne diseases in Africa based on environmental data have received considerable attention in recent years and are tools with public health potential (*10*,*15*). The mechanisms by which environmental factors influence meningitis epidemics in Africa are unclear (*3*). Areas within the traditional meningitis belt and beyond, however, are environmentally susceptible to epidemics with potentially large populations at risk; markers such as absolute humidity and dust profiles, land-cover type, and population density are independent predictors of these areas. In addition, rainfall and dust are predictors in some, but not all regions, and the potential to develop region-specific models that could be more sensitive within given ecologic zones warrants further study. Our findings could facilitate the development of models to identify regions with increased vulnerability to epidemics in the future and provide a basis for monitoring the impact of climate variability and environmental change on epidemic occurrence in Africa.
